# Potential Therapeutic Roles of Tanshinone IIA in Human Bladder Cancer Cells

**DOI:** 10.3390/ijms150915622

**Published:** 2014-09-04

**Authors:** Sheng-Chun Chiu, Sung-Ying Huang, Shu-Fang Chang, Shee-Ping Chen, Chi-Cheng Chen, Tien-Huang Lin, Hsin-Ho Liu, Tsung-Hsun Tsai, Shang-Sen Lee, Cheng-Yoong Pang, Teng-Fu Hsieh

**Affiliations:** 1 Department of Research, Taichung Tzu Chi Hospital, Buddhist Tzu Chi Medical Foundation, No. 88, Section 1, Fengxing Road, Tanzi Dist., Taichung 427, Taiwan; E-Mails: honeyhopes@gmail.com (S.-C.C.); fantac10@gmail.com (S.-F.C.); 2 Department of Ophthalmology, Mackay Memorial Hospital, No. 690, Section 2, Guangfu Road, East Dist., Hsinchu 30071, Taiwan; E-Mail: hopes929@yahoo.com.tw; 3 Tzu Chi Stem Cells Center, Hualien Tzu Chi Hospital, Buddhist Tzu Chi Medical Foundation, No. 707, Section 3, Chung-Yang Road, Hualien 970, Taiwan; E-Mail: asdinap@yahoo.com.tw; 4 Department of Urology, Taichung Tzu Chi Hospital, Buddhist Tzu Chi Medical Foundation, No. 88, Section 1, Fengxing Road, Tanzi Dist., Taichung 427, Taiwan; E-Mails: kukoc0925@gmail.com (C.-C.C.); thlin@hotmail.com (T.-H.L.); thlin@hotmail.com (H.-H.L.); zoneshin@yahoo.com.tw (T.-H.T.); j520037@yahoo.com.tw (S.-S.L.); 5 School of Medicine, Tzu Chi University, No. 701, Section 3, Chung-Yang Road, Hualien 970, Taiwan; 6 Department of Medical Research, Hualien Tzu Chi Hospital, Buddhist Tzu Chi Medical Foundation, No. 707, Section 3, Chung-Yang Road, Hualien 970, Taiwan; 7 Institute of Medical Sciences, School of Medicine, Tzu Chi University, No. 701, Section 3, Chung-Yang Road, Hualien 970, Taiwan

**Keywords:** Tanshinone IIA, bladder cancer, metastasis, cisplatin, combination therapy

## Abstract

Tanshinone IIA (Tan-IIA), one of the major lipophilic components isolated from the root of *Salviae Miltiorrhizae*, has been found to exhibit anticancer activity in various cancer cells. We have demonstrated that Tan-IIA induces apoptosis in several human cancer cells through caspase- and mitochondria-dependent pathways. Here we explored the anticancer effect of Tan-IIA in human bladder cancer cell lines. Our results showed that Tan-IIA caused bladder cancer cell death in a time- and dose-dependent manner. Tan-IIA induced apoptosis through the mitochondria-dependent pathway in these bladder cancer cells. Tan-IIA also suppressed the migration of bladder cancer cells as revealed by the wound healing and transwell assays. Finally, combination therapy of Tan-IIA with a lower dose of cisplatin successfully killed bladder cancer cells, suggesting that Tan-IIA can serve as a potential anti-cancer agent in bladder cancer.

## 1. Introduction

Bladder cancer (BCa) ranks sixth in cancer incidence in the United States [1]. Approximately 30% of newly-diagnosed superficial BCa are multifocal initially, 60%–70% of superficial BCa will recur, and 10%–20% of them will undergo stage progression to muscle-invasive or metastatic disease [2]. Superficial BCa are treated by surgical resection and intravesical (within the bladder) Bacille Calmette-Guérin (BCG) immunotherapy with 5-year survival rate approaches 90%, but for invasive BCa with radical cystectomy and systemic chemotherapy, at least 50% of these BCa patients will still die from metastases within 2 years of diagnosis, and the treatment fails in 95% of patients with less than 10% 5-year survival rate for the metastatic BCa [3]. The high recurrence rate of BCa has raised the cost of treatment from $96,000 to $187,000 per BCa patient (from diagnosis to death) [4], and with an estimated total cost of $3.98 billion in 2010 [5].

**Figure 1:**
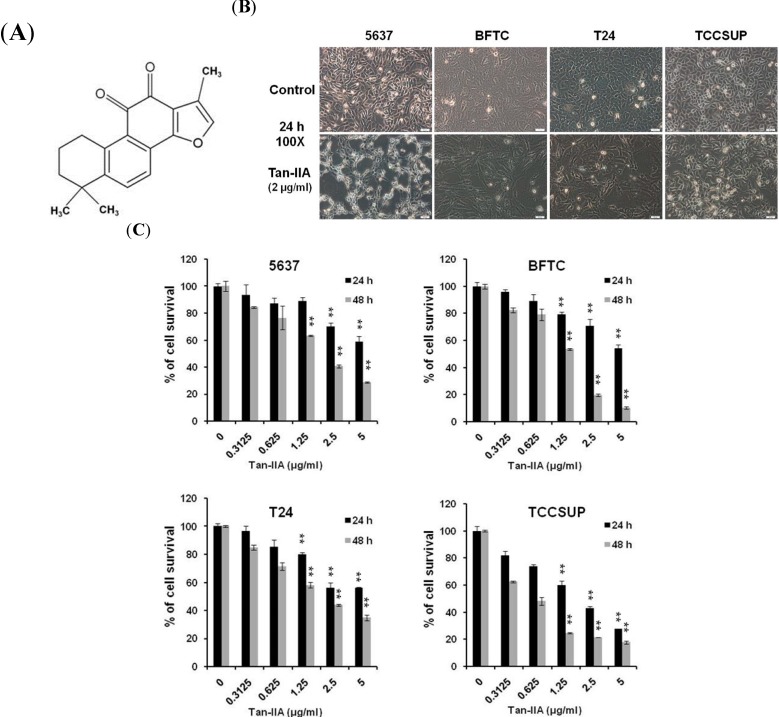
Effects of Tan-IIA on the viability of human bladder cancer cells. (**A**) Chemical structure of Tan-IIA, C_19_H_18_O_3_, molecular weight: 293.34; (**B**) Human bladder cancer cells (5637, BFTC, T24 and TCCSUP) were treated with 0.2% DMSO as control or 2 μg/mL Tan-IIA for 24 h, were shown; Scale bar: 50 μm; (**C**) Human bladder cancer cells were treated with increasing concentration of Tan-IIA (0.3125 to 5 μg/mL) for 24 (black bar) and 48 h (gray bar), respectively, and the survival rate was evaluated with MTT assay. Data are presented as means ± S.D. obtained from three different experiments. ** *p* < 0.01 *versus* vehicle.

Tanshinone IIA (Tan-IIA, Figure 1A) is an extract from a widely used traditional Chinese medicine, Danshen (*Salvia miltiorrhiza*) and its antitumor activity in numerous human cancer cell types has been well documented [6,7,8,9,10]. The antitumor activity of Tan-IIA is mainly due to proliferation inhibition and apoptosis induction [11,12,13]. Induction of endoplasmic reticulum (ER) stress has also been noted [10,14,15]. Tan-IIA also decreases human cancer cells invasion and metastasis [16,17,18,19]. Thus, Tan-IIA can serve as a potential anti-cancer agent in cancer therapy. However, no investigation on Tan-IIA and bladder cancer has been disclosed to date. We thus conducted a series of experiments to investigate the effect of Tan-IIA on bladder cancer cells.

## 2. Results

### 2.1. Tan-IIA Inhibited Cell Proliferation in Human Bladder Cancer Cells

To determine the cytotoxicity effect and the optimized dosage of Tan-IIA in bladder cancer cells, cells were treated with increasing concentrations of Tan-IIA for 24 and 48 h, respectively, and subsequently evaluated by the MTT assay. As shown in Figure 1B, all 4 bladder cancer cell lines (5637, BFTC, T24, TCCSUP) shrank at 24 h after Tan-IIA treatment as compared to the untreated cells. By using the MTT assay, we further demonstrated that Tan-IIA significantly decreased the viability of various bladder cancer cell lines in a dose- and time-dependent manner (Figure 1C) Treatment of 5637 cells with 2.5 μg/mL Tan-IIA for 24 and 48 h resulted in 70.3% and 40.7% cell survival, respectively. Treatment of BFTC cells with 2.5 μg/mL Tan-IIA for 24 and 48 h resulted in 70.6% and 19.7% cell survival, respectively. Treatment of T24 cells with 2.5 μg/mL Tan-IIA for 24 and 48 h resulted in 56.3% and 43.8% cell survival, respectively. Treatment of TCCSUP cells with 2.5 μg/mL Tan-IIA for 24 and 48 h resulted in 43% and 21.3% cell survival, respectively. The IC_50_ at 48 h of Tan-IIA treatment in bladder cancer cells were: 5637, 2.6 μg/mL; BFTC, 2 μg/mL; T24, 2.7 μg/mL; TCCSUP, 1.4 μg/mL, respectively.

### 2.2. Tan-IIA Induced Sub-G1 Population Accumulation in Human Bladder Cancer Cells

**Figure 2:**
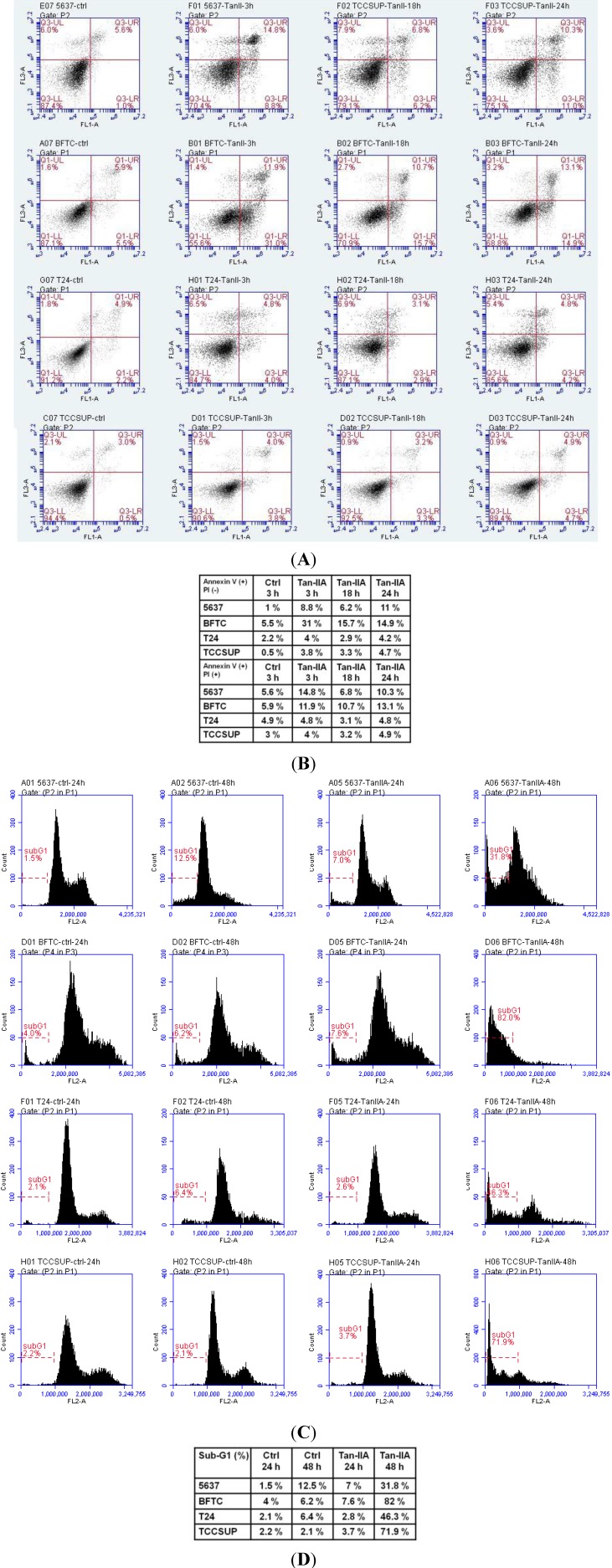
Flow cytometric analysis of bladder cancer cells treated with Tan-IIA. (**A**) Human bladder cancer cells were analyzed by annexin V-FITC staining in the vehicle group for 3 h or in the presence of 4 μg/mL Tan-IIA for 3, 18 and 24 h, respectively; (**B**) The percentage of annexin V-FITC positive population in bladder cancer cells in (**A**) is shown; (**C**) The accumulation of sub-G1 cell population in the presence or absence of 4 μg/mL Tan-IIA for 24 and 48 h, respectively; (**D**) The percentage of sub-G1 population in bladder cancer cells in (**C**) is shown.

To evaluate the role of apoptosis in Tan-IIA-induced bladder cancer cell death, flow cytometric analysis and annexin V-FITC staining was performed (Figure 2). Human bladder cancer cells treated with 4 μg/mL Tan-IIA for the indicated time points were analyzed by flow cytometry (Figure 2A). The annexin V-FITC positive populations increased after Tan-IIA treatment as compared to the vehicle group (Figure 2B). Early apoptosis was noted as early at 3 h after Tan-IIA treatment. The appearance of cell population in the Sub-G1 phase can be considered as the degree of apoptotic cell death. As shown in Figure 2C, the addition of 4 μg/mL Tan-IIA resulted in the increased accumulation of cells in the sub-G1 phase. The sub-G1 population increased to 31.8% (5637), 82% (BFTC), 46.3% (T24) and 71.9% (TCCSUP), respectively, after Tan-IIA treatment for 48 h (Figure 2D). 

### 2.3. Tan-IIA Induced Mitochondria Dependent Apoptosis in Human Bladder Cancer Cells

**Figure 3:**
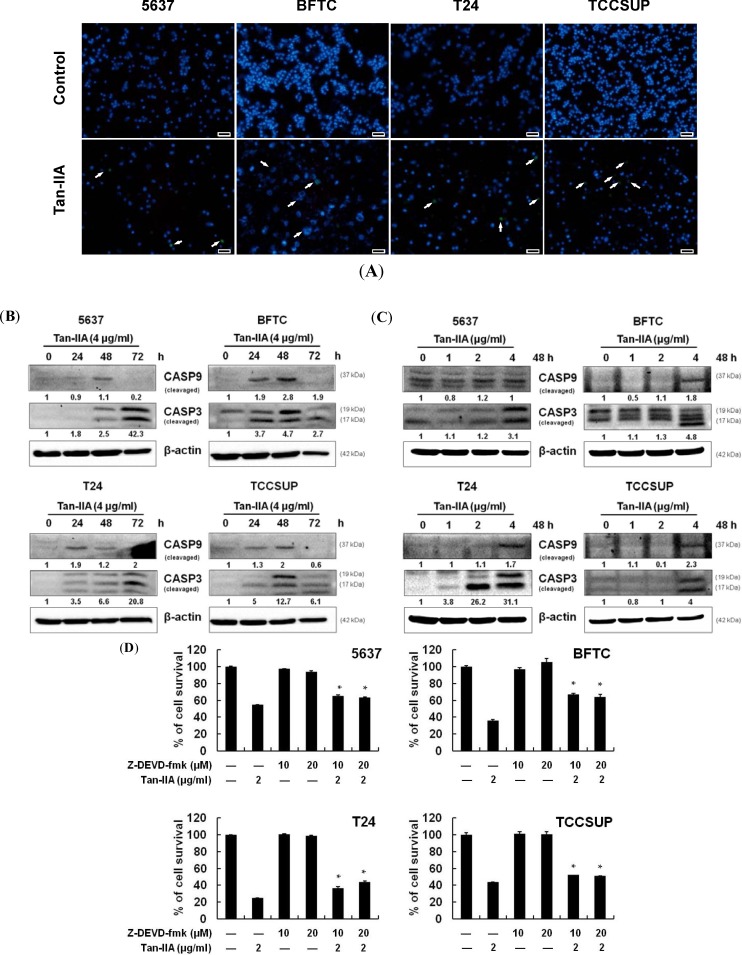
Tan-IIA induced mitochondria dependent apoptosis in human bladder cancer cells. (**A**) Human bladder cancer cells were treated with 0.2% DMSO (control) or 2 μg/mL Tan-IIA for 72 h and then were fixed and stained with the TUNEL assay. Nuclei were stained with DAPI. TUNEL positive cells were indicated by arrows. Scale bar: 50 μm; (**B**) Human bladder cancer cells were treated with 4 μg/mL Tan-IIA for 0 to 72 h, and western blot analysis were performed for cleaved caspase 9 and cleaved caspase 3. β-actin was used as an internal control; (**C**) Human bladder cancer cells were treated with increasing concentration of Tan-IIA (1 to 4 μg/mL) for 48 h, followed by western blot analyses for cleaved caspase 9 and cleaved caspase 3. β-actin was used as an internal control; (**D**) MTT assay of human bladder cancer cells pretreated with caspase 3 inhibitor Z-DEVD-fmk (10 or 20 μM) for 1 h and then treated with or without 2 μg/mL Tan-IIA for 48 h. The values are the mean ± S.D. from three independent experiments. * *p* < 0.05 *versus* vehicle.

To further investigate how Tan-IIA induced bladder cancer cell death, the TUNEL staining was performed. Cells treated with 2 μg/mL Tan-IIA for 72 h were collected and stained with the TUNEL staining kit. The late stage of apoptosis was observed by TUNEL-positive cells compared with untreated cells (Figure 3A). Activation of caspase family proteins is the crucial events for apoptosis. Among them, caspase-9 and -3 are key cysteine-protease associated with mitochondria-dependent apoptosis. Cleavages of caspase-9 and -3 increased time- (Figure 3B) and dose- (Figure 3C) dependently in bladder cancer cells treated with Tan-IIA. However, the activation of caspase 8 (*i.e.*, the extrinsic apoptotic cell death) was not noted (*data not shown*). These data suggest that bladder cancer cells may undergo mitochondria-dependent apoptosis after expose to Tan-IIA. To examine whether caspase 3 is involved in Tan-IIA-induced apoptosis, cells were pretreated with caspase 3 inhibitor Z-DEVD-fmk (10 or 20 μM) for 1 h and then treated with or without 2 μg/mL Tan-IIA for 48 h (Figure 3D). The caspase 3 inhibitor partly blocked Tan-IIA-induced cell death (65.4% and 64.4% in 5637, 67.2% and 63.7% in BFTC, 36.6% and 43.7% in T24 and 52.4% and 50.9% in TCCSUP) as compared to the Tan-IIA alone group (54.6% in 5637, 36% in BFTC, 24.7% in T24, 43.8% in TCCSUP). These data suggest that Tan-IIA-induced cell death is mediated through a caspase-dependent pathway.

### 2.4. Effect of Tan-IIA on the Migration of Human Bladder Cancer Cells

**Figure 4:**
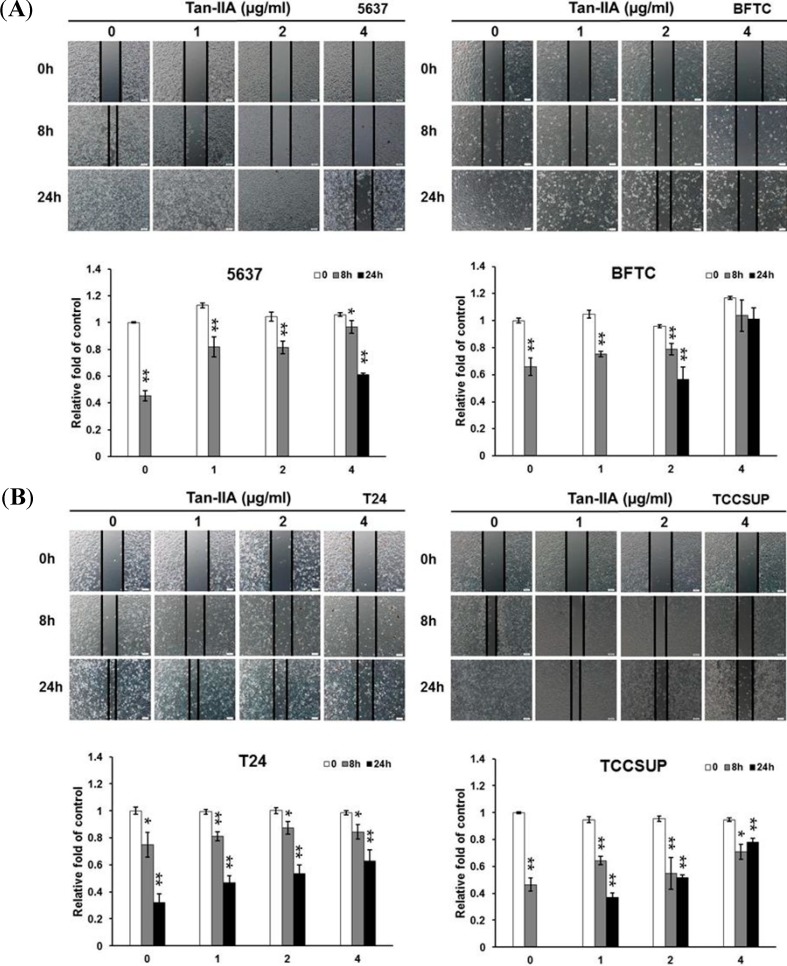
Effect of Tan-IIA on the migration of human bladder cancer cells-wound healing experiment. (**A**) Human bladder cancer cells (5637, BFTC) were treated with 0.2% DMSO as the control or 1 to 4 μg/mL Tan-IIA for the indicated time points; Scale bar: 100 μm; (**B**) Human bladder cancer cells (T24, TCCSUP) were treated with 0.2% DMSO as the control or 1 to 4 μg/mL Tan-IIA for the indicated time points. Images of wound closures were captured using inverted microscope with 100× magnification; Scale bar: 100 μm. The cell-free area invaded by migrated cells across the black lines were calculated by three randomized fields and quantified. The cell-free distance at 0 h were set at as 100%. Data are from three different experiments and presented as means ± S.D. * *p* < 0.05; ** *p* < 0.01 *versus* vehicle.

Wound closure was examined at 0, 8 and 24 h, respectively in the presence of various amount of Tan-IIA (0 to 4 μg/mL). As shown in Figure 4, the non-treated cells migrated into the scratched area and filled the gap at 24 h. The migration of Tan-IIA-treated bladder cancer cells was inhibited, especially in BFTC cells. The extents of inhibition of migration by 4 μg/mL Tan-IIA at 24 h for 5637, BFTC, T24 and TCCSUP were 60.9%, 100.2%, 63% and 77.8%, respectively. These data suggest that the migration ability of human bladder cancer cells was inhibited by Tan-IIA treatment in a dose- and time-dependent manner.

**Figure 5:**
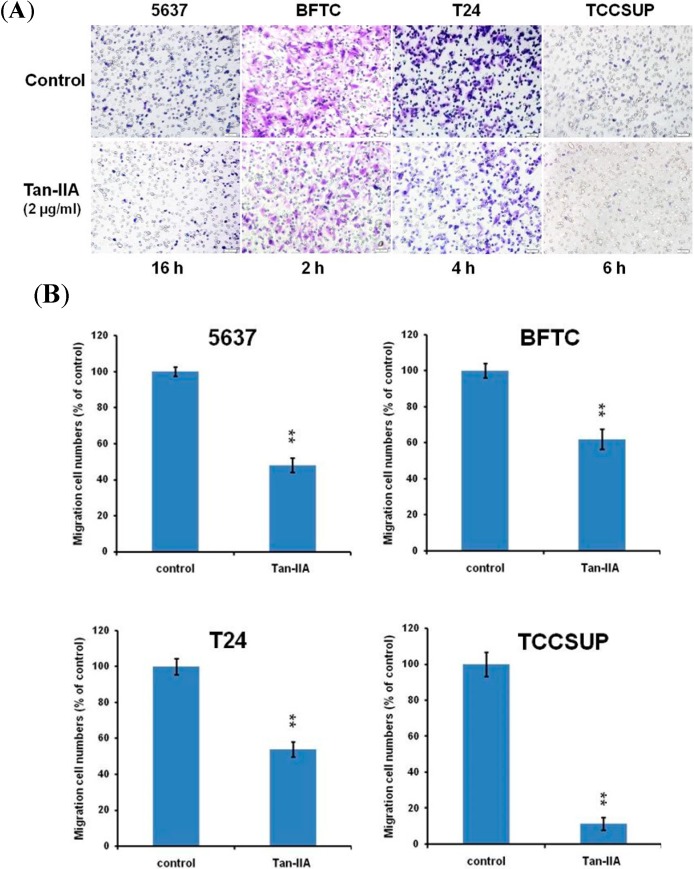
Effect of Tan-IIA on the migration of human bladder cancer cells-transwell test. (**A**) human bladder cancer cells were pretreated with 0.2% DMSO as control or 2 μg/mL Tan-IIA for 24 h and then seeded onto the transwell hanging insert for different time points (5637: 16 h, BFTC: 2 h, T24: 4 h and TCCSUP: 6 h). Images were captured using an inverted microscope with 200× magnification; Scale bar: 50 μm; (**B**) The migration of human bladder cancer cells was quantified by enumerating the stained cells that migrated into the underside of the hanging insert membrane. Data are presented as means ± S.D. from three different experiments. ** *p* < 0.01 *versus* vehicle.

In transwell analysis, the migrated Tan-IIA-treated (2 μg/mL) human bladder cancer cells decreased significantly as compared to the untreated cells (Figure 5A). The migrated cancer cells accounted for 48.12% (5637), 61.88% (BFTC), 53.85% (T24) and 11.44% (TCCSUP), respectively, compared to the non-treated cells (Figure 5B).

### 2.5. Effect of Tan-IIA on the Combination Therapy with Cisplatin

**Figure 6:**
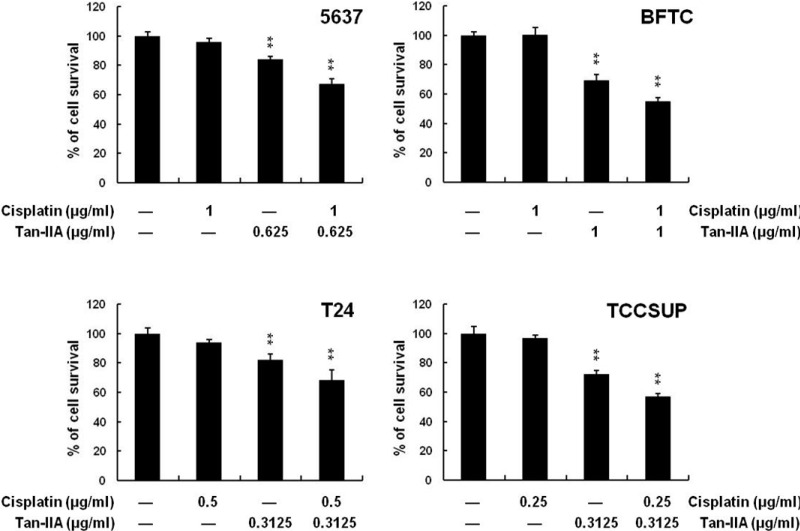
Effect of Tan-IIA on combination therapy with cisplatin. Human bladder cancer cells were treated with cisplatin or Tan-IIA only, or a combination therapy for 24 h, and analyzed with MTT assay. Data are presented as means ± S.D. from three different experiments. ** *p* < 0.01 *versus* vehicle.

Human bladder cancer cells were treated with only cisplatin or Tan-IIA, or a combination therapy for 24 h, and then analyzed with the MTT assay. Tan-IIA showed synergic effects with cisplatin combination therapy: 5637: 67.51% (cisplatin: 96.1%; Tan-IIA: 83.95%), BFTC: 55.14% (cisplatin: 100%; Tan-IIA: 69.13%), T24: 67.51% (cisplatin: 93.84%; Tan-IIA: 82.35%) and TCCSUP: 57.25% (cisplatin: 96.93%; Tan-IIA: 72.39%) (Figure 6).

## 3. Discussion

Several dietary compounds including resveratrol, gingerol, and curcumin have been demonstrated to induce apoptosis in various cancer cells [20]. The mechanisms of Tan-IIA cytotoxicity include anti-proliferation, apoptosis induction and ER stress induction in various cancer cell lines [15,21,22,23,24,25,26]. Recent studies have demonstrated that Tan-IIA is cytotoxic to bladder cancer [27,28]. By using various bladder cancer cell lines, we delineated the mechanism of how Tan-IIA killed these bladder cancer cells. All of the cell lines showed sensitivity to Tan-IIA treatment in a time- and dose-dependent manner, especially the TCCSUP cells.

Further analysis revealed that the cause of death is probably apoptosis as revealed by increasing sub-G1 population in flow cytometric analysis, and was later confirmed by Annexin V-FITC staining. Besides, our results demonstrated that the mitochondria-dependent pathway was involved in Tan-IIA-induced apoptosis in these human bladder cancer cells: Tan-IIA induced caspase-9 and caspase-3 cleavages in a time- and dose-dependent manner. Pretreatment of caspase 3 inhibitor Z-DEVD-fmk significantly rescued these cells from Tan-IIA cytotoxicity. Similarly, Tan-IIA also induced apoptosis via the mitochondria-dependent pathway in lung, colon and prostate cancer cells in our previous studies [10,25,29]. The late-stage apoptosis was revealed by the TUNEL staining. Thus, increased levels of caspase-9 and -3 activation appeared to correlate with mitochondria-dependent apoptosis in Tan-IIA-induced bladder cancer cell death.

The malignancy of tumors cells usually correlates to their proliferation and invasion. The increasing motility and invasiveness are also the key features of metastasis [30]. Szarvas *et al.* showed that Tan-IIA inhibited MMP2 and MMP9 expression, which in turn decreased tumor aggressiveness and invasiveness [31]. The migration of human bladder cancer cell lines was suppressed by Tan-IIA treatment in our study, which indicated the anti-metastatic effect of Tan-IIA on bladder cancer cells as well. However, the exact mechanism of Tan-IIA on migration inhibition, as well as anti-metastasis, needs further investigation.

Chemotherapy is used to increase lifespan and prevent disease recurrence following surgical resection of localized cancer. Chemotherapy is also utilized as part of the multimodal treatment of locally advanced or metastatic cancer, allowing more limited surgery with organ sparing and even cure. Therefore, more effective and less toxic chemotherapy regimens are likely to significantly benefit cancer patients. Cisplatin has been previously used in chemotherapy regimens for patients with urothelial cell carcinoma [32,33].

Although chemotherapy remains the main option for cancer therapy, urothelial carcinoma cells of bladder usually develop chemoresistance. Only modest response rates are obtained using multi-agents regimens including cisplatin [33]. In our present study, after treated with Tan-IIA these bladder cancer cells demonstrated increase sensitivity to cisplatin. Resistance to chemotherapy affects drug efficacy. The mechanisms of drug resistance include drug inactivation, alterations in drug target, processing of drug-induced damage, and evasion of apoptosis [34,35]. In ovary cancer cell, Tan-IIA induced apoptosis, reduced cisplatin resistance in COC1/DDP cells, and caused significant growth inhibition through p38-mediated down-regulation of survivin, ERCC1 and LRP mRNA expression [36]. In order to develop Tan-IIA as a potential drug in multi-agents regimen, it will be of great importance to delineate the mechanism of Tan-IIA in altering cancer cell sensitivity to cisplatin or others in the future.

## 4. Experimental Section

### 4.1. Cell Culture

The human bladder cancer cell lines 5637, T24 and TCCSUP were purchased from ATCC (American Type Culture Collection, Manassas, VA, USA). The human bladder cancer cell line BFTC (BFTC 905) was purchased from BCRC (Bioresource Collection and Research Center, Hsinchu, Taiwan). Cells were cultured in appropriate medium (5637, BFTC and T24: RPMI 1640, TCCSUP: DMEM) supplemented with 10% heat-inactivated fetal bovine serum, 100 U/mL penicillin and 100 U/mL streptomycin (all from Invitrogen, Carlsbad, CA, USA) at 37 °C in a humidified atmosphere with 5% CO_2_.

### 4.2. Chemicals and Antibodies

Tanshinone IIA (C_19_H_18_O_3_, >97% HPLC), Cisplatin, Dimethyl sulfoxide (DMSO), [3-(4,5-dimethyl thizol-2-yl)-2,5-diphenyl tetrazolium bromide] (MTT), crystal violet, DSD, Tween-20, methanol, Z-DEVD-fmk and horseradish peroxidase-conjugated secondary antibodies were purchased from Sigma Chemical Co. (St. Louis, MO, USA). The antibody against cleaved caspase-3 (Asp175), cleaved caspase-9 and β-actin were all purchased from Cell Signaling Technology, Inc., Danvers, MA, USA). Polyvinyldenefluoride (PVDF) membranes, BSA protein assay kit and western blot chemiluminescence reagent were purchased from Amersham Biosciences (Arlington Heights, IL, USA).

### 4.3. Western Blot Analysis

Five hundred thousand cells per 6-cm plate were lysed with 200 µL M-PER mammalian protein extraction reagent containing protease inhibitor cocktail (Thermo Scientific, Rockford, IL, USA) and centrifuged at 13,000× *g* at 4 °C for 10 min. The protein concentration in the supernatants was quantified using a BSA Protein Assay Kit. Electrophoresis was performed on a NuPAGE Bis-Tris Electrophoresis System using 20 µg of reduced protein extract per lane. Resolved proteins were transferred to PVDF membranes, blocked with 5% skim milk for 1 h at room temperature, finally probed with the specific primary antibodies at 4 °C overnight. After the PVDF membrane was washed three times with TBS/0.2% Tween-20 at room temperature, it was incubated with appropriate secondary antibody labeled with horseradish peroxidase (goat anti-mouse or anti-rabbit, 1:10,000, Sigma Chemical, St. Louis, MO, USA) for 1 h at room temperature. All resolved proteins bands were detected using Western Lightning™ Chemiluminescence Reagent Plus (Amersham Biosciences, Arlington Heights, IL, USA).

### 4.4. MTT Assay

The viability of the cells after treatment with Tan-IIA was evaluated using the MTT assay preformed in triplicate. Briefly, cells (4 × 10^4^/well) were incubated in 24-well plates containing 0.5 mL of serum-containing medium. Cells were allowed to adhere for 18–24 h and were washed with phosphate-buffered saline (PBS). Solutions were always prepared fresh by dissolving 0.2% DMSO (control) or Tan-IIA in culture medium before their addition to cells. The Tan-IIA-containing medium was removed after treatment for 24 or 48 h, cells were washed with PBS, and culture medium containing 300 μg/mL MTT was added for 1 h at 37 °C. After the MTT medium was removed, 0.5 mL of DMSO was added to each well. Absorbance at 570 nm was detected by a PowerWave X Microplate ELISA Reader (Bio-Tek Instruments, Winooski, VT, USA). The absorbance for DMSO-treated cells was considered as 100%.

### 4.5. TUNEL Assay

Human bladder cancer cells were cultured in the presence or absence of Tan-IIA (2 μg/mL) for 72 h and then examined for apoptosis with the TUNEL assay (*In Situ* Cell Death Detection Kit, Roche, Taiwan).

### 4.6. Annexin V-FITC Staining

Human bladder cancer cells were cultured in the presence of Tan-IIA (4 μg/mL) for 3, 18 and 24 h. The vehicle control groups were treated with 0.2% DMSO. Apoptotic cell death was examined using annexin V-FITC detection kits according to the manufacturer’s instructions (BD Biosciences, San Diego, CA, USA). Ten thousand events were acquired for each sample and analyzed by the Accuri C6 flow cytometer with CFlow^®^ software.

### 4.7. Flow Cytometric Analysis

The cell cycle was determined by flow cytometry following DNA staining to reveal the total amount of DNA. Approximately 5 × 10^5^ of bladder cancer cells were incubated with 4 μg/mL Tan-IIA for the indicated time. Cells were harvested with trypsin/EDTA, collected, washed with PBS, fixed with cold 100% ethanol overnight, and then stained with a solution containing 20 μg/mL PI, 0.2 mg/mL RNase A, and 0.1% Triton X-100 for 30 min in the dark. The cells were then passed through an Accuri C6 flow cytometer to measure the DNA content. The data were obtained and analyzed with CFlow^®^ software.

### 4.8. Cell Migration Assay

Migration was determined using the wound healing and the transwell assay. Wound healing assay: cells were seeded and grown overnight to 90%~95% confluence in 24-well plates (5637: 3 × 10^4^, BFTC: 7 × 10^3^, T24: 1.5 × 10^4^, TCCSUP: 2.5 × 10^4^). Migration was tested in wound-healing assays using culture inserts (ibidi, Martinsried, Germany). Cells were washed with PBS and cultured with medium containing 0 to 4 μg/mL Tan-IIA. Wound closure was evaluated and photographed at 0, 8 and 24 h with an inverted microscope (Olympus CKX41 fluorescence microscope, Melville, NY, USA).

The transwell assay was performed using hanging inserts (Millipore Co., Billerica, MA, USA) in 24-well plates. Cells were seeded (5 × 10^4^) in the hanging inserts, which were filled with culture medium or with medium supplemented with 2 μg/mL Tan-IIA. Culture medium supplemented with 10% FBS was added in the bottom chamber. Incubation was carried out at 37 °C for the indicated time points (5637: 16 h; BFTC: 2 h; T24: 4 h; TCCSUP: 6 h). The hanging inserts were washed with PBS, and cells on the upper filter surface were wiped away with a cotton swab. The inserts were subsequently fixed with 10% formalin for 10 min at room temperature, stained with 0.2% *w*/*v* crystal violet, washed with PBS, and counted under a light microscope operating at 200× magnification. The migration cell numbers of the control group were considered as 100%.

### 4.9. Statistical Analysis

All data are shown as mean ± S.D. Statistical differences were analyzed using the Student’s *t*-test for normally distributed values and by nonparametric Mann–Whitney *U*-test for values with a non-normal distribution. Significant differences between groups were evaluated using analysis of variance (ANOVA) with Games-Howell test as *post-hoc* test.

## 5. Conclusions

In conclusion, our study demonstrated that Tan-IIA causes cytotoxicity and induces apoptosis in human bladder cancer cells. The major underlying mechanism is the activation of caspases-9 and caspase-3. The anti-metastatic effect of Tan-IIA in human bladder cancer cells was shown by wound healing and transwell migration assay. The synergic effects of Tan-IIA in combination with cisplatin were also shown. Taken together, our findings indicate that Tan-IIA triggers apoptosis in human bladder cancer cells through the mitochondria-dependent apoptotic pathway, and may become a potential antitumor compound for bladder cancer therapy.
